# Determination of the regulatory network and function of the lysR-type transcriptional regulator of *Lactiplantibacillus plantarum*, *Lp*LttR

**DOI:** 10.1186/s12934-022-01774-9

**Published:** 2022-04-20

**Authors:** Xin-Xin Liu, Lei Liu, Xin Song, Guang-Qiang Wang, Zhi-Qiang Xiong, Yong-Jun Xia, Lian-Zhong Ai

**Affiliations:** Shanghai Engineering Research Center of Food Microbiology, School of Health Science and Engineering, University of Shanghai for Science and Technology, Shanghai, 200093 China

**Keywords:** *Lactiplantibacillus plantarum*, LysR type transcriptional regulator (LttR), Transcriptional regulation, Regulon

## Abstract

**Background:**

*Lactiplantibacillus plantarum* has various healthcare functions including the regulation of immunity and inflammation, reduction of serum cholesterol levels, anti-tumor activity, and maintenance of the balance of intestinal flora. However, the underlying metabolic and regulatory mechanisms of these processes remain unclear. Our previous studies have shown that the LysR type transcriptional regulator of *L. plantarum* (*Lp*LttR) regulates the biotransformation of conjugated linoleic acids (CLAs) through the transcriptional activation of *cla-dh* (coding gene for CLA short-chain dehydrogenase) and *cla-dc* (coding gene for CLA acetoacetate decarboxylase). However, the regulatory network and function of *Lp*LttR have not yet been characterized in *L. plantarum*.

**Results:**

In this study, the regulatory role of *Lp*LttR in various cellular processes was assessed using transcriptome analysis. The deletion of *Lp*LttR had no evident influence on the bacterial growth. The transcriptome data showed that the expression of nine genes were positively regulated by *Lp*LttR, and the expression of only two genes were negatively regulated. Through binding motif analysis and molecular interaction, we demonstrated that the regulatory region of the directly regulated genes contained a highly conserved sequence, consisting of a 15-base long box and rich in AT.

**Conclusion:**

This study revealed that *Lp*LttR of *L. plantarum* did not play a global regulatory role similar to that of the other transcriptional regulators in this family. This study broadens our knowledge of *Lp*LttR and provides a theoretical basis for the utilization of *L. plantarum*.

## Background

The LysR type transcriptional regulatory factor (LttR) is a ubiquitous regulatory factor in prokaryotes. Many studies have shown that this family of regulators plays a regulatory role in many cellular processes including primary metabolism, secondary metabolism, stress response, cell division, toxicity, quorum sensing and protection, etc. [1, 2]. In *Pseudomonas putida* KT2440, CatR regulates the expression of *catBCA,* which is involved in the catechol gene cluster [3]. The CbnR in *Ralstonia eutropha* regulates the transcription of the catechol explanation related gene cluster *cbnABCD *[4]. The TfdR of *R. eutropha* JMP134 not only controls the expression of the o-phenylene diene gene cluster *tfdDCEFB*, but also regulates the transcription of *tfdA* to metabolize 2,4-dichlorophenoxyacetic acid isooctyl ester [5]. Moreover, LttRs regulate the genes involved in virulence, metabolism, quorum sensing and exercise [1]. As more target genes have been identified, LttRs have been identified as global regulators.

LttRs are structurally conserved, with most containing 276–324 amino acid residues. Their N-terminal is a typical DNA binding domain named the HTH domain, and the C-terminal is the substrate or co-inducer binding domain, which is less conservative and is convenient for the recognition and binding of substrates [6]. LttRs are often induced or suppressed by small molecules, and usually form dimers or tetramers to activate or inhibit the expression of target genes [1].

*L. plantarum* is a common lactic acid bacteria (LAB) used in the production of several functional and fermented foods [7]. However, there have been few studies on its metabolism and regulatory mechanisms. Our previous studies have shown that *L. plantarum* ATCC BAA-793 can convert linoleic acid (LA) to conjugated linoleic acid (CLA), which plays an important role in reducing weight and regulating immunity. The LysR family transcriptional regulator, *Lp*LttR, activates the transcription of *cla-dh* and *cla-dc* during this process and promotes the biosynthesis of CLA [8]. In addition to *cla-dh* and *cla-dc*, it is still unknown which genes are regulated by *Lp*LttR and the biological processes in which it participates. With continuous study of prokaryotic transcriptional regulation, more target genes of LttRs have been identified. However, the regulatory mechanisms in different species remain to be clarified, especially in *L. plantarum,* which has a wide range of application prospects in the food industry.

Here, we studied the regulon of *Lp*LttR of *L. plantarum* ATCC BAA-793*.* The sequence analysis of *Lp*LttR was performed to predict its function. We then performed transcriptome sequencing of the WT and *LplttR* knockout mutant strains (Δ*LplttR*) to identify the differentially expressed genes. Moreover, the presumed binding sites of the target genes were predicted and verified using molecular interactions based on interferometric techniques. The findings of this study revealed the regulatory network of *Lp*LttR in *L. plantarum* and provided new insights into the functions of LttRs*.*

## Results

### *Lp*LttR conservative analysis

**Fig. 1:**
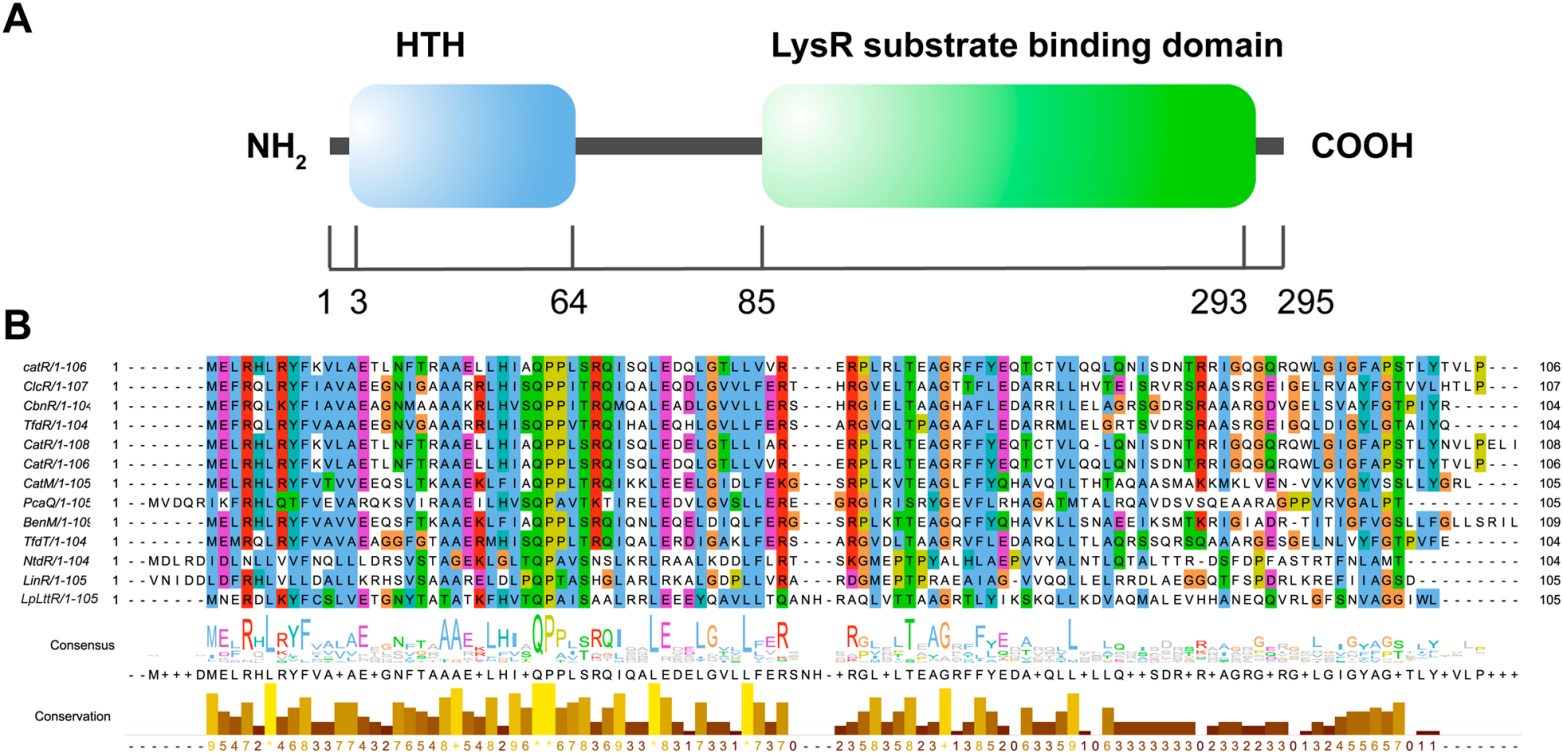
*Lp*LttR conservative analysis. **A** The illustration of the domain organization of *Lp*LttR. *Lp*LttR was composed of two domains: one HTH domain (position 3–64) at the N-terminal and one LysR substrate-binding domain (position 85–293) at the C-terminal. **B** Sequence conservatism analysis of the HTH domain of LttRs including CatR (*Pseudomonas putida KT2440*)*,* ClcR (*P. putida*), CbnR (*Ralstonia eutropha*), TfdR (*R. eutropha* JMP134), CatR (*P. putida*), CatM (*Acinetobacter*) PcaQ (*Agrobacterium*), BenM (*Acinetobacter*), TfdT (*Burkholderia*), NtdR (*Acidovorax*), LinR (*Sphingomonas*), and *Lp*LttR (*Lactob*acillus *plantarum*)

The primary structure of *Lp*LttR in *L. plantarum* ATCC BAA-793 was analyzed in this study. It was encoded by *LP_RS00230* and composed of 295 amino acids. Pfam domain analysis showed that *Lp*LttR contained an HTH domain and a LysR substrate binding domain at the C and N terminals at residues 3–64 and 85–293, respectively (Fig. 1A). The amino acid sequence of the HTH domain of *Lp*LttR was aligned with other LttRs which have been extensively studied and reported as CatR in *P. putida* KT2440 [4], ClcR in *P. putida* [9], TcbR in *Pseudomonas* sp. strain P51 [10], CbnR in *R. eutropha* [11], TfdR in *R. eutropha* JMP134 [12], CatR in *P. putida PaW85* [13], CatR in *P. putida* PRS1 [4], TfdR in *Ralstonia* [12], TfdS in *Ralstonia* [5], CatM in *Acinetobacter *[14], PcaQ in *Agrobacterium *[15]*,* BenM in *Acinetobacter *[16]*,* TfdT in *Burkholderia *[12]*,* NtdR in *Acidovorax *[17]*,* and LinR in *Sphingomonas *[18]. The sequence alignment showed a high sequence conservation between the HTH domain of *Lp*LttR and other LttRs, especially for the first 50 amino acid residues (Fig. 1B). This suggests that *Lp*LttR might also directly bind to DNA regulatory regions and play a regulatory role in a variety of cellular processes, similar to other LttRs.

### LpLttR knockout strain construction

**Fig. 2:**
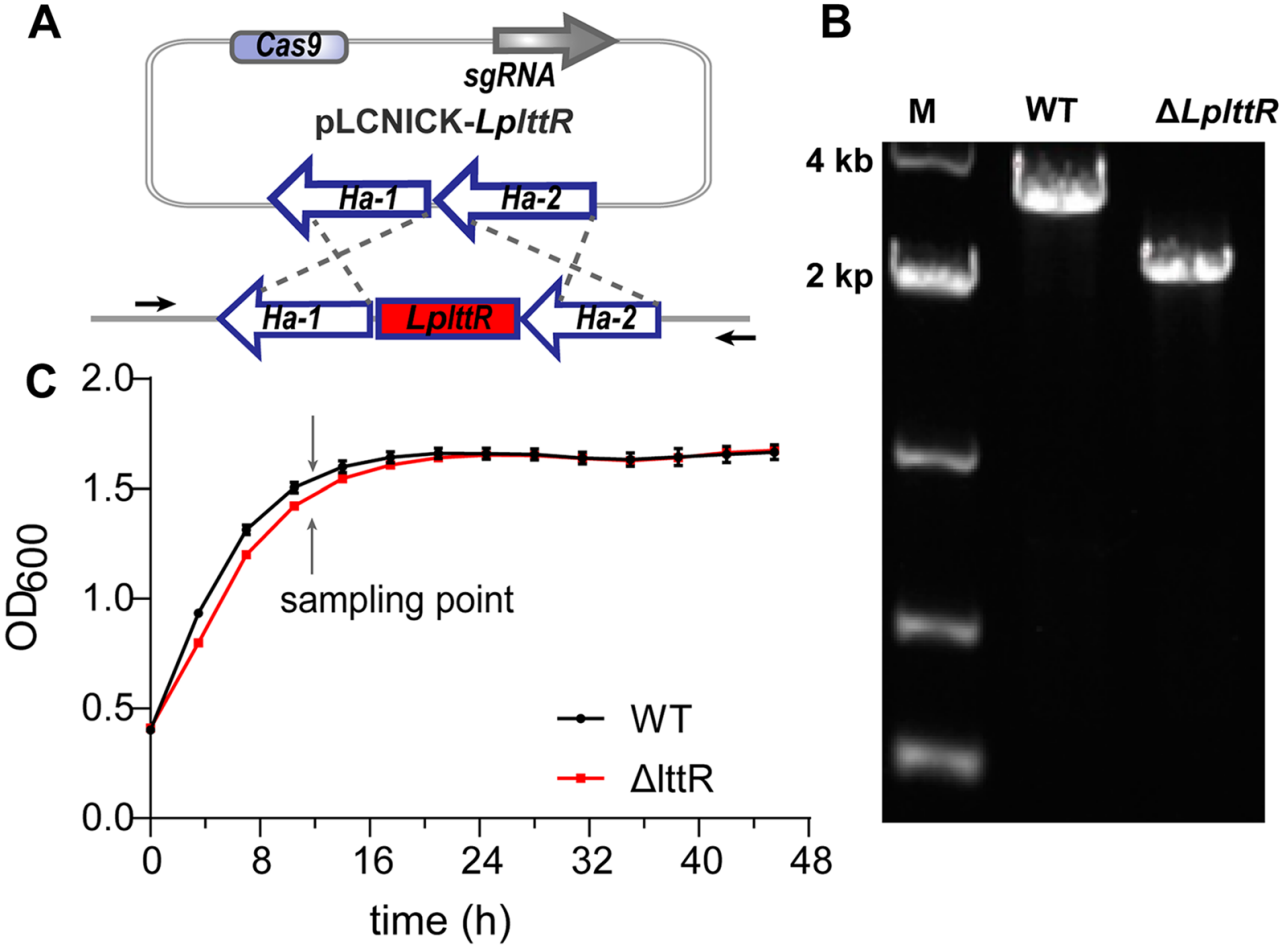
Construction of the *LplttR* knockout strain and its effects of on bacterial growth. **A** Schematic diagram of the *LplttR* knockout principle. *Ha-1* and *Ha-2* represent the upstream and downstream homologous arms respectively. The black arrows indicate the location of colony PCR primers used for verification. **B** Identification of *LplttR* knockout by colony PCR; **C** Effect of the *LpLttR* mutation on bacterial growth. Error bars indicate the standard deviations of three independent experiments

To study the function of *Lp*LttR, we constructed the knockout strain(Δ*LplttR*) based on CRISPR-Cas9 gene editing technology. As shown in Fig. 2A, the upstream and downstream 1000 bp of *LplttR* were selected as the upstream and downstream homologous arms (Ha-1 and Ha-2), respectively. The homologous arms and sgRNA were inserted into the pLCNICK knockout plasmid. Verification primers were designed on both sides of the homologous arms to verify the transformers using colony PCR. As shown in Fig. 2B, the amplified product of the putative knockout strain (Δ*LplttR*) was approximately 1000 bp smaller than that of the WT, suggesting that the *LplttR* gene was deleted successfully. To study the effect of *LplttR* on bacterial growth, the growth curves of WT and Δ*LplttR* strains were investigated. As shown in Fig. 2C, during the first 24 h, the *ΔLplttR* strain grew slower than the WT but not thereafter.

### Identification of the *Lp*LttR regulon

**Fig. 3:**
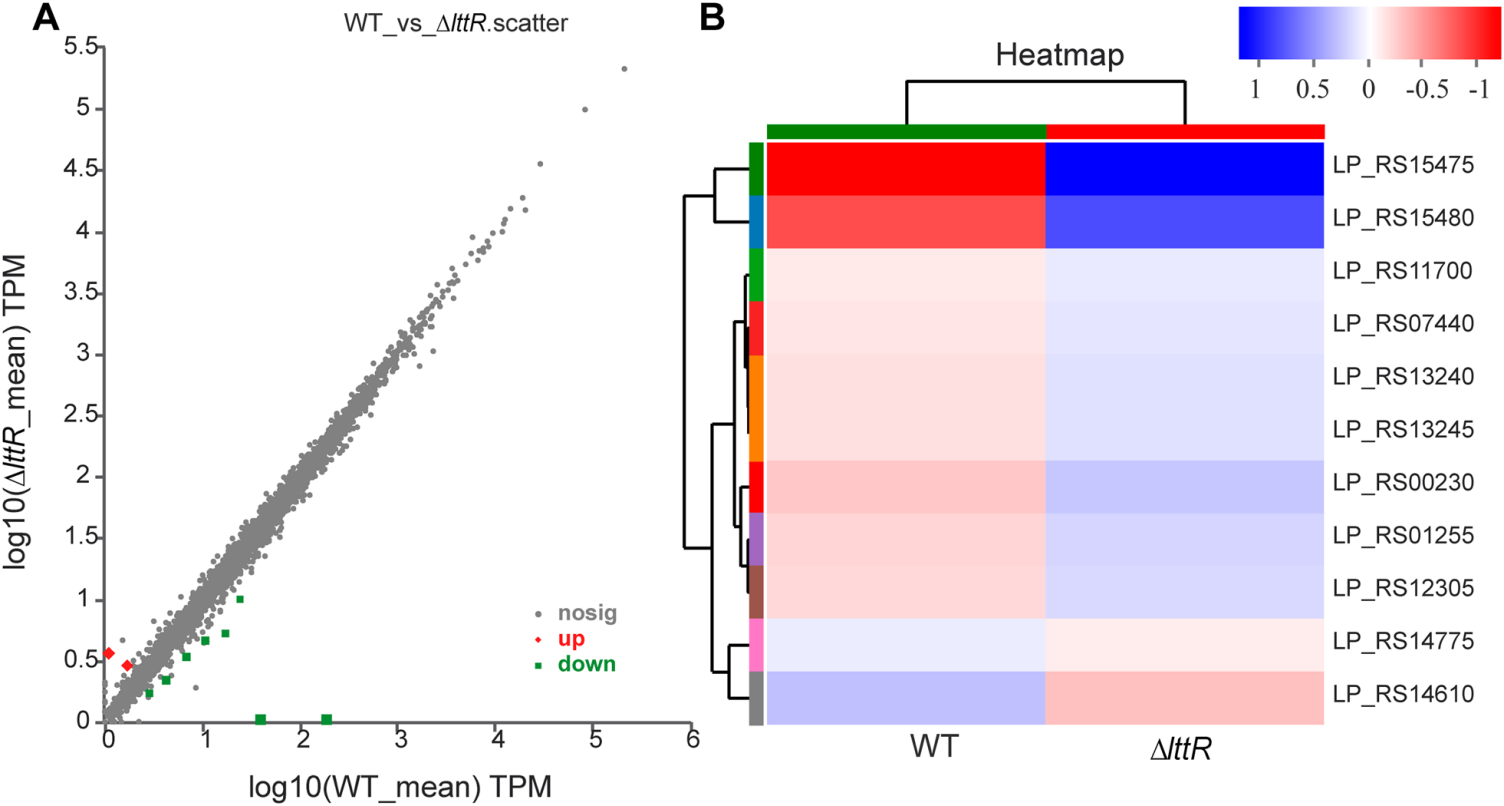
Analysis of the target genes with more than twofold difference in expression. **A** Scatter plot of the differentially expressed genes. The X-axis and Y-axis represent the expression of genes in the WT and *LplttR* knockout strains. The values are all log-transformed. Each dot in the image represents a specific gene. The red dots indicate the up-regulated genes and green dots indicate the down-regulated genes. **B** Heatmap of the differentially expressed genes. Each column represents different strains and each row represents a gene. The depth of the color in the heatmap indicates the amount of the gene expression. For the specific amount of the expression, please see the digital label on the color bar

As the main family of transcriptional regulators in prokaryotes, LttRs regulate a variety of genes and even play a global regulatory role in *P. aeruginosa*, *S. thermophilus*, and *Salmonella enterica *[19–21]. To identify the regulons of *Lp*LttR in *L. plantarum*, we analyzed the mRNA expression levels of WT and Δ*LplttR.* As shown in Fig. 3A, in Δ*LplttR*, there were 11 mRNAs with a fold difference of more than two- fold, of which the expression of nine genes were down-regulated. Among the differentially expressed genes, *LP_RS15475* and *LP_RS15480* expressions were down regulated most substantially (Fig. 3B), and were described as replication proteins and hypothetical proteins, respectively. The expression of *LP_RS14775* and *LP_RS14610,* separately annotated as SLC45 family MFS transporter and IS1182 family transposase, were upregulated. The transcriptional levels of these 11 genes were analyzed using RT-qPCR. Although the genes differed with respect to fold-change, the trend of up-regulation and down-regulation was similar, thus, confirming the reliability of the transcriptome data. All of the differentially expressed genes and their descriptions are listed in Table 1. Base on the number and function of the differential genes, we inferred that unlike other LttRs, *Lp*LttR did not play a global regulatory role in *L. plantarum*.

**Table 1:** Differential expressed genes with the difference more than twofold

Gene name	Gene description	FC(ΔlttR/WT)
*LP_RS00230*	LysR family transcriptional regulator	0.291
*LP_RS15475*	replication protein	0
*LP_RS15480*	hypothetical protein	0
*LP_RS14775*	SLC45 family MFS transporter	2.198
*LP_RS14610*	IS1182 family transposase	100.747
*LP_RS13240*	cell wall hydrolase/muramidase	0.46
*LP_RS11700*	MFS transporter	0.443
*LP_RS13245*	SH3 domain-containing protein	0.47
*LP_RS07440*	ABC transporter permease	0.44
*LP_RS01255*	LysM peptidoglycan-binding domain-containing protein	0.423
*LP_RS12305*	APC family permease	0.436

**Fig. 4:**
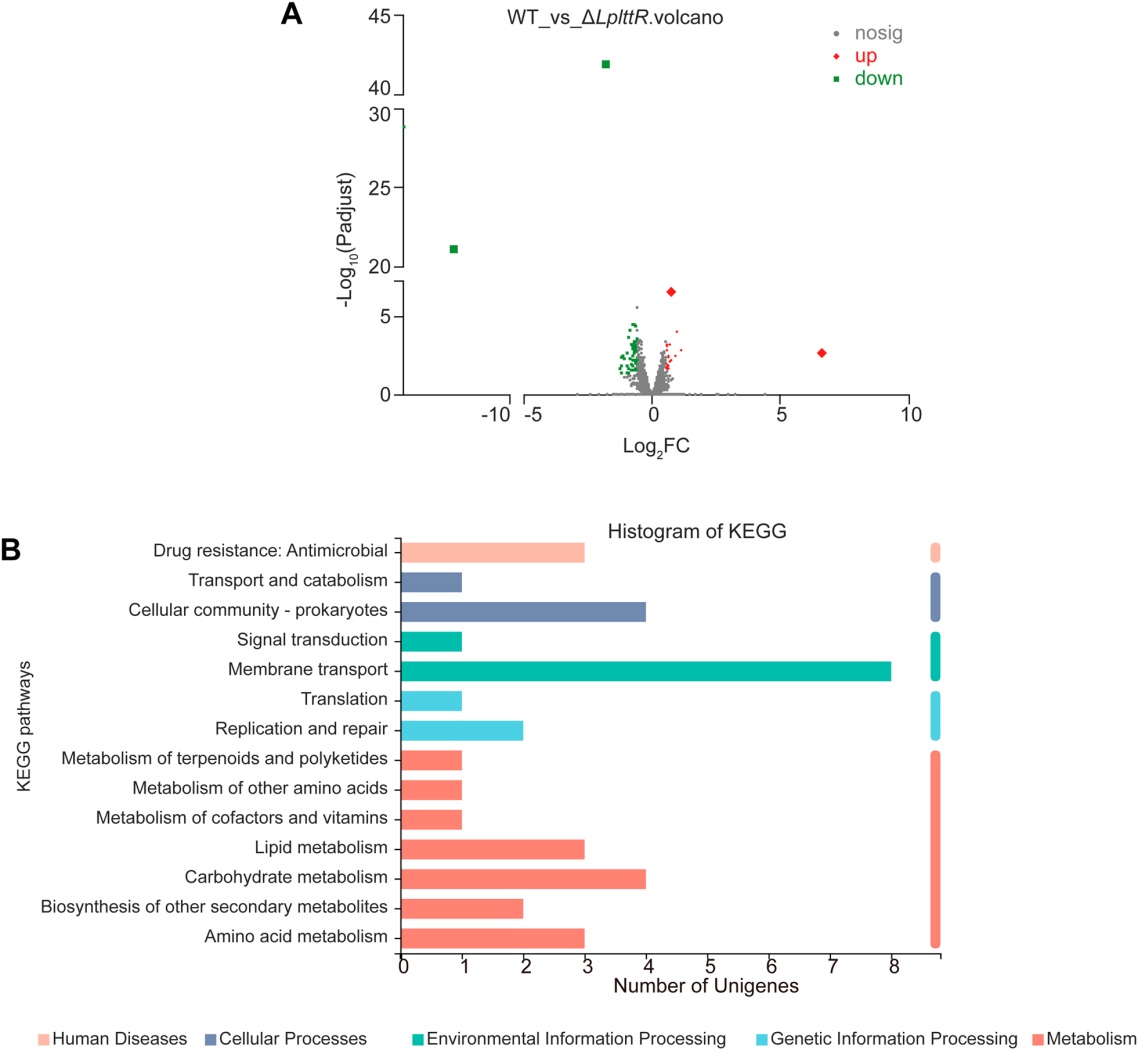
Analysis of the target genes with more than 1.5-fold difference. **A** Volcano diagram of the *Lp*LttR regulon. Abscissa is the multiple change value of the difference of gene expression between the WT and *LplttR* knockout strains; namely the FC value. The ordinate is the statistical test value of the difference of gene expression, P value. Note the logarithmic scale of horizontal and vertical coordinates. Each dot in the picture represents a specific gene. **B** Functional annotation analysis of target genes. The ordinate is the name of the KEGG metabolic pathway, and the abscissa is the number of genes annotated to the pathway

To further analyze the regulatory role of *Lp*LttR in *L. plantarum*, we also analyzed the differentially expressed genes with a change of more than 1.5-fold. As shown in Table 2, there were 70 genes with a fold difference of more than 1.5-fold, with a P-value < 0.05. *Lp*LttR mainly acted as a transcriptional activator, since 49 of the 70 differential genes were down-regulated*,* whereas the remaining 21 genes were up-regulated in Δ*LplttR* (Fig. 4A). The 70 differentially expressed genes were assigned to different groups based on KEGG pathway analysis. As shown in Fig. 4B, 12 genes participated in metabolism, nine in environmental information processing, three in genetic information processing, and three in human disease. Notably, eight of the environmental information processing genes were carbon transport and sensing related proteins, that were closely correlated with carbohydrate metabolism, especially the fatty acid metabolism (Table 3). The PTS sugar transporters (*agaF* and *celB*), galactose mutarotase (*galM*), glycoside hydrolase (*bglA*), fatty acid metabolism-related enzymes (*fabD, fabZ, fabH*), and several ABC transporter proteins (*oppA, agaF, fliY, oppC, metI,* and *oppD*) were under the control of *Lp*LttR, suggesting that *Lp*LttR might play an important role in carbohydrate metabolism in *L. plantarum*.

**Table 2:** Differential genes identified by transcriptome analysis

Gene name	Gene description	Type	Regulate	FC(ΔlttR/WT)
*LP_RS14610*	IS1182 family transposase	mRNA	Up	100.747
*LP_RS14775*	SLC45 family MFS transporter	mRNA	Up	2.198
*LP_RS15415*	DUF916 and DUF3324 domain-containing protein	mRNA	Up	1.935
*LP_RS01010*	DUF871 domain-containing protein	mRNA	Up	1.931
*LP_RS11220*	GntR family transcriptional regulator	mRNA	Up	1.702
*LP_RS12660*	Glycoside hydrolase family 1 protein	mRNA	Up	1.7
*LP_RS01000*	PTS sugar transporter subunit IIC	mRNA	Up	1.696
*LP_RS13760*	Hemolysin III family protein	mRNA	Up	1.613
*treR*	Trehalose operon repressor	mRNA	Up	1.611
*LP_RS01020*	DgaE family pyridoxal phosphate-dependent ammonia lyase	mRNA	Up	1.572
*LP_RS02110*	Serine transporter	mRNA	Up	1.57
*spx*	Transcriptional regulator Spx	mRNA	Up	1.541
*LP_RS10520*	Hypothetical protein	mRNA	Up	1.535
*LP_RS14565*	Galactose mutarotase	mRNA	Up	1.532
*LP_RS05750*	Aspartate-semialdehyde dehydrogenase	mRNA	Up	1.52
*LP_RS06040*	Hypothetical protein	mRNA	Up	1.512
*LP_RS15420*	WxL domain-containing protein	mRNA	Up	1.512
*LP_RS11200*	PTS sugar transporter subunit IIA	mRNA	Up	1.506
*LP_RS12525*	DUF916 and DUF3324 domain-containing protein	mRNA	Up	1.503
*LP_RS05765*	NADH-dependent flavin oxidoreductase	mRNA	Up	1.503
*LP_RS00610*	Helix-turn-helix transcriptional regulator	mRNA	Up	1.5
*LP_RS05365*	Peptide ABC transporter substrate-binding protein	mRNA	Down	0.668
*LP_RS12020*	LysM peptidoglycan-binding domain-containing protein	mRNA	Down	0.668
*LP_RS13890*	HIT family protein	mRNA	Down	0.663
*LP_RS01675*	AraC family transcriptional regulator	mRNA	Down	0.659
*LP_RS00140*	Hypothetical protein	mRNA	Down	0.656
*LP_RS00290*	ASCH domain-containing protein	mRNA	Down	0.655
*LP_RS06330*	Response regulator transcription factor	mRNA	Down	0.654
*LP_RS13055*	ABC transporter ATP-binding protein	mRNA	Down	0.646
*LP_RS06150*	DUF916 and DUF3324 domain-containing protein	mRNA	Down	0.643
*LP_RS06145*	LPXTG cell wall anchor domain-containing protein	mRNA	Down	0.643
*LP_RS05380*	ABC transporter ATP-binding protein	mRNA	Down	0.642
*mvk*	Mevalonate kinase	mRNA	Down	0.642
*LP_RS07140*	ACP S-malonyltransferase	mRNA	Down	0.637
*LP_RS07130*	Ketoacyl-ACP synthase III	mRNA	Down	0.636
*LP_RS05375*	ABC transporter permease	mRNA	Down	0.633
*LP_RS11365*	LysR family transcriptional regulator	mRNA	Down	0.629
*LP_RS07625*	GIY-YIG nuclease family protein	mRNA	Down	0.625
*fabZ*	3-hydroxyacyl-ACP dehydratase FabZ	mRNA	Down	0.623
*LP_RS05800*	Membrane protein	mRNA	Down	0.619
*LP_RS12295*	Helix-turn-helix transcriptional regulator	mRNA	Down	0.615
*LP_RS04070*	MucBP domain-containing protein	mRNA	Down	0.611
*LP_RS04675*	AraC family transcriptional regulator	mRNA	Down	0.609
*LP_RS08765*	Hypothetical protein	mRNA	Down	0.609
*LP_RS13480*	Transporter substrate-binding domain-containing protein	mRNA	Down	0.606
*LP_RS14300*	C40 family peptidase	mRNA	Down	0.603
*LP_RS07570*	Ribonuclease HI family protein	mRNA	Down	0.601
*LP_RS12415*	Zinc ribbon domain-containing protein	mRNA	Down	0.597
*LP_RS12410*	Zinc-ribbon domain-containing protein	mRNA	Down	0.59
*LP_RS11005*	Viroplasmin family protein	mRNA	Down	0.589
*LP_RS11130*	Hypothetical protein	mRNA	Down	0.583
*LP_RS11350*	Aminotransferase class I/II-fold pyridoxal phosphate-dependent enzyme	mRNA	Down	0.576
*LP_RS12030*	Methylated-DNA–[protein]-cysteine S-methyltransferase	mRNA	Down	0.571
*LP_RS15375*	2-keto-4-pentenoate hydratase	mRNA	Down	0.558
*LP_RS02570*	AEC family transporter	mRNA	Down	0.544
*LP_RS08575*	ISL3 family transposase	mRNA	Down	0.532
*LP_RS13185*	YxeA family protein	mRNA	Down	0.528
*LP_RS13235*	Ldh family oxidoreductase	mRNA	Down	0.524
*LP_RS12675*	LysM peptidoglycan-binding domain-containing protein	mRNA	Down	0.521
*LP_RS08270*	GNAT family N-acetyltransferase	mRNA	Down	0.511
*LP_RS00810*	Peptide ABC transporter substrate-binding protein	mRNA	Down	0.51
*LP_RS13245*	SH3 domain-containing protein	mRNA	Down	0.47
*LP_RS13240*	Cell wall hydrolase/muramidase	mRNA	Down	0.46
*LP_RS11700*	MFS transporter	mRNA	Down	0.443
*LP_RS07440*	ABC transporter permease	mRNA	Down	0.44
*LP_RS12305*	APC family permease	mRNA	Down	0.436
*LP_RS01255*	LysM peptidoglycan-binding domain-containing protein	mRNA	Down	0.423
*LP_RS00230*	LysR family transcriptional regulator	mRNA	Down	0.291
*LP_RS15475*	Replication protein	mRNA	Down	0
*LP_RS15480*	Hypothetical protein	mRNA	Down	0

**Table 3:** Differential genes involved in carbohydrate metabolism, lipid metabolism, and environmental information processing in KEGG pathway analysis

Gene ID	Description	KO Name	KO Description
*Carbohydrate metabolism*			
*LP_RS11200*	PTS sugar transporter subunit IIA	agaF	PTS system, N-acetylgalactosamine-specific IIA component [EC:2.7.1.-]
*LP_RS14565*	galactose mutarotase	galM	aldose 1-epimerase [EC:5.1.3.3]
*LP_RS12660*	glycoside hydrolase family 1 protein	bglA	6-phospho-beta-glucosidase [EC:3.2.1.86]
*LP_RS01000*	PTS sugar transporter subunit IIC	celB	PTS system, cello
*Lipid metabolism*			
*LP_RS07140*	ACP S-malonyltransferase	fabD	[acyl-carrier-protein] S-malonyltransferase [EC:2.3.1.39]
*fabZ*	3-hydroxyacyl-ACP dehydratase FabZ	fabZ	3-hydroxyacyl-[acyl-carrier-protein] dehydratase [EC:4.2.1.59]
*LP_RS07130*	ketoacyl-ACP synthase III	fabH	3-oxoacyl-[acyl-carrier-protein] synthase III [EC:2.3.1.180]
*Environmental information processing*			
*LP_RS05365*	peptide ABC transporter substrate-binding protein	oppA	oligopeptide transport system substrate-binding protein
*LP_RS13055*	ABC transporter ATP-binding protein		iron complex transport system ATP-binding protein [EC:3.6.3.34]
*LP_RS11200*	PTS sugar transporter subunit IIA	agaF	PTS system, N-acetylgalactosamine-specific IIA component [EC:2.7.1.-]
*LP_RS13480*	transporter substrate-binding domain-containing protein	fliY	L-cystine transport system substrate-binding protein
*LP_RS05375*	ABC transporter permease	oppC	oligopeptide transport system permease protein
*LP_RS07440*	ABC transporter permease	metI	D-methionine transport system permease protein
*LP_RS05380*	ABC transporter ATP-binding protein	oppD	oligopeptide transport system ATP-binding protein
*LP_RS01000*	PTS sugar transporter subunit IIC	celB	PTS system, cellobiose-specific IIC component
*LP_RS06330*	response regulator transcription factor	nreC	two-component system, NarL family, response regulator NreC

### Preliminary exploration of the regulatory mechanism of *Lp*LttR

**Fig. 5:**
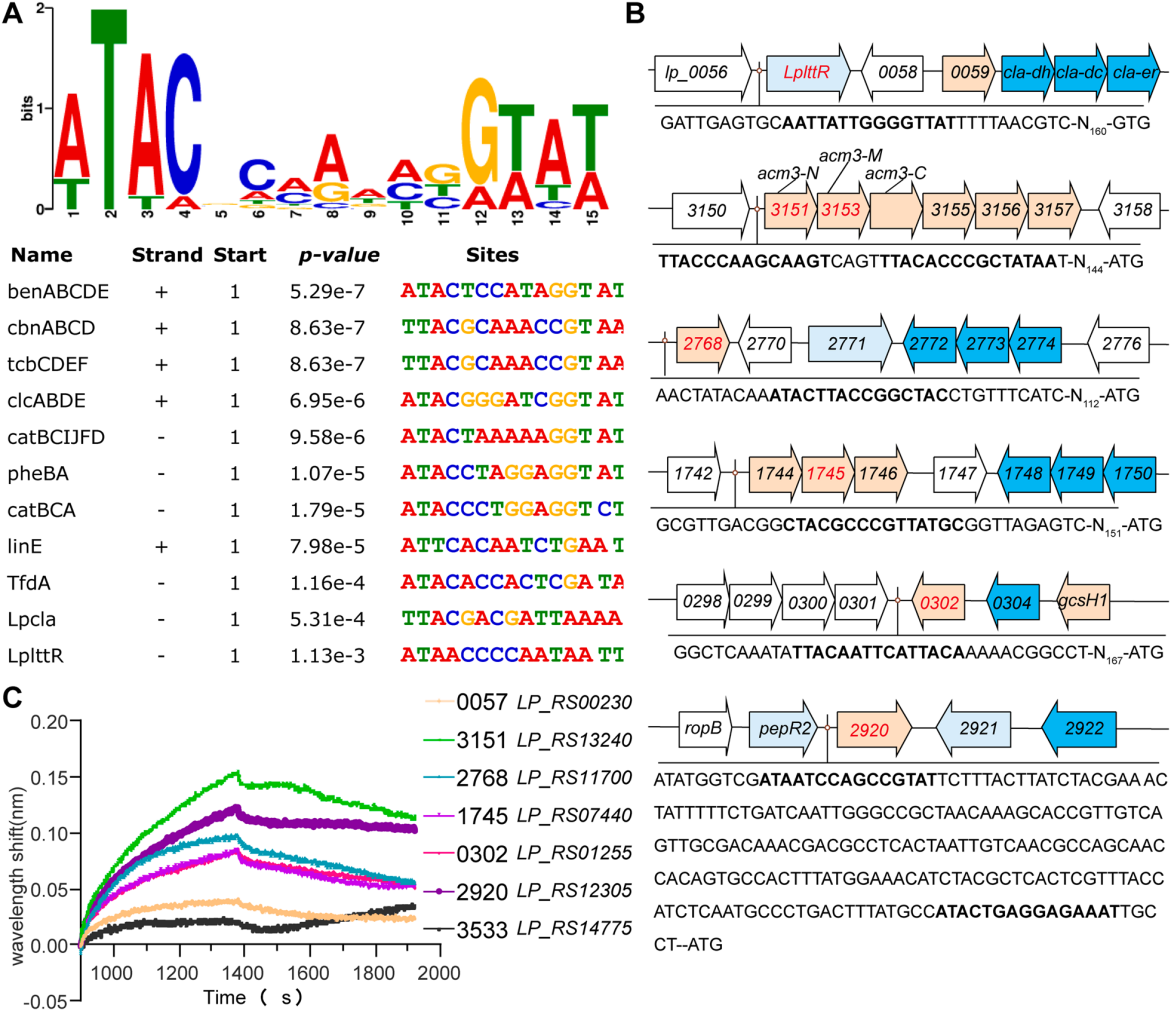
*Lp*LttR binding sites analysis. **A** Detecting the conserved binding motif of *Lp*LttR using the MEME online tool. The LttRs used as the MEME input included the *LpLttR* and *cla* operon regulated by LpLttR in *Lactobacillus plantarum, catBCA* regulated by CatR in *Pseudomonas putida, clcABD* operon on plasmid pAC27 regulated by ClcR, *tcbCDEF* on plasmid pP51 controlled by TcbR of *Pseudomonas* sp. strain P51, *cbnABCD* controlled by CbnR in *Ralstonia eutropha, tfdA* regulated by TfdR/S in *R. eutropha* JMP134, catBCIJFD regulated by CatM in *Acinetobacter* sp. *benABCDE* controlled by BenM in *Acinetobacter*, and *linE-linD* regulated by *LinR* in *Sphingomonas.* The motif count setting was searching for one motif. Motif width was between 6 and 50. **B** The predicted binding sites of *LplttR* on the target gene promoters. The operons of the differential genes were predicted by the website (http://www.microbesonline.org/operons/gnc220668.html). **C** Molecular interaction of *Lp*LttR to the regulatory region of the target genes. Both the correspondence of the gene names annotated in KEGG database and that used in the transcriptome sequencing are listed. The interaction mainly contains two processes: association and dissociation. During the association process, the spectral interference shift increased, while the wavelength shift decreased during dissociation. Different colors represent different target gene promoters

To better understand the sequence characteristics of the LpLttR binding box, a MEME search was performed to analyze the conserved motifs. The binding motif of *Lp*LttR was predicted using MEME (https://meme-suite.org/meme/) according to the binding sites reported in previous studies, including the *LplttR* and *cla* operon regulated by *Lp*LttR in *L. plantarum* [8], *catBCA* regulated by CatR in *P. putida* [4], *clcABD* operon on plasmid pAC27 regulated by ClcR [9], *tcbCDEF* on plasmid pP51 controlled by TcbR of *Pseudomonas* sp. strain P51 [10], *cbnABCD* controlled by CbnR in *R. eutropha* [22], *tfdA* regulated by TfdR/S in *R. eutropha* JMP134 [5], catBCIJFD regulated by CatM in *Acinetobacter* sp. *benABCDE* controlled by BenM in *Acinetobacter*, and *linE-linD* regulated by *LinR* in *Sphingomonas *[23]*.* Default settings were used in the site distribution (zero or one occurrence per sequence (zoops)). As shown in Fig. 5A, the predicted motif of *Lp*LttR possessed a conserved binding motif 5′- (A/T)TAC-n_7_-(G/A)( T/A) a(T/A) -3′.

Next, the regulatory regions of the differentially expressed genes that increased or decreased more than twice were analyzed using bioinformatics*.* As shown in Fig. 5B, the regulatory regions of *LP_RS00230*, *LP_RS13240* (located in the same transcription unit with *LP_RS13245)*, *LP_RS11700*, *LP_RS07440*, *LP_RS01255,* and *LP_RS12305* contained the potential LttR binding motif, suggesting that the promoter of these genes might be directly bound to *Lp*LttR. *LP_RS15475* and *LP_RS15480* were located on plasmids p0203 and p0203, respectively. The cis-element analysis of these two genes was not performed.

To further verify whether *Lp*LttR regulates the expression of these genes directly, we used the purified *Lp*LttR-His protein and the regulatory region DNA of the target genes to carry out molecular interaction experiments. *LP_RS14775,* with no predicted LttR-binding motif, was used as the negative control. As shown in Fig. 5C, *Lp*LttR had the strongest affinity to the promoter of *LP_RS12305* (KD = 0.104 uM) and different degrees of binding with *LP_RS00230*, *LP_RS13240, LP_RS11700*, *LP_RS07440*, and *LP_RS01255,* but not with *LP_RS14775*, which was consistent with the results of the binding site analysis. The specific binding between *Lp*LttR and its own promoter region suggested that *Lp*LttR in *Lactiplantibacillus plantarum* was self-regulated, similar to other LttRs. These results suggested that *LP_RS00230, LP_RS15475*, *LP_RS15480, LP_RS11700, LP_RS07440*, and *LP_RS12305* were directly regulated by *Lp*LttR in *L. plantarum.*

## Discussion

*L. plantarum* was one of the best studied *lactobacilli*; hundreds of studies and dozens of reviews have described its metabolism and regulation of metabolism. In our previous study, we found that the CLA biotransformation was transcriptionally regulated by *Lp*LttR. However, the regulon of *Lp*LttR in this species remained uncharacterized. In this study, we identified the regulon of *Lp*LttR by transcriptomic analysis of the WT and *LplttR* knockout strains. *Lp*LttR was highly conserved in sequence, and its knockout caused the transcriptional difference of 70 genes to be more than 1.5-fold, and 11 genes to be more than twofold. Many of the differentially expressed genes were mapped to the perception, metabolism, and transportation of carbon sources, revealing that *LplttR* might perform important functions in carbon metabolism. Through bioinformatics analysis and molecular interactions, we further verified that *LpLttR* directly regulated the expression of *LP_RS00230, LP_RS13240*, *LP_RS11700, LP_RS07440*, *LP_RS01255,* and *LP_RS12305* by binding to the promoter regions. The binding motif consisted of a highly conserved consensus sequence: 5′-(A/T)TAC-N_7_-(G/A)( T/A) a(T/A)-3′.

Our previous study showed that *Lp*lttR responded to LA and activated the transcription of *cla-dh* and *cla-dc*, promoting the biotransformation of CLA [8]. However, the *Lp*lttR regulon identified in this study does not contain *cla-dh* and *cla-dc*. This may be due to the difference in the culture media and conditions. The medium used in this study did not contain LA, under which condition the transcription of *cla-dh* and *cla-dc* was suppressed. According to previous studies, LttRs are often induced or suppressed by environmental or metabolic co-inducers, thus activating or inhibiting the expression of target genes. For example, the metabolic intermediates of aromatic compounds generally act as inducers of LttR in the regulation aromatic compound metabolism-related genes. It has even been shown that BenM could bind to different co-inducers leading to an altered protein conformation [1]. Therefore, the cellular processes in which LpLttR participates and its regulon may vary under different culture conditions.

## Conclusions

In summary, we investigated the function and regulation of *Lp*lttR in *L. plantarum.* The sequence of *Lp*LttR was highly conserved with that of other transcriptional regulators in this family. However, the knockout of *Lp*LttR showed no significant effect on the bacterial growth. Coinciding with the growth, only 11 genes exhibited a more than twofold transcriptional increase. *LP_RS00230, LP_RS13240*, *LP_RS11700, LP_RS07440*, *LP_RS01255,* and *LP_RS12305* were directly regulated by *Lp*LttR. *Lp*LttR regulated the transcription of these genes by binding to the conserved LttR box (5′- (A/T)TAC-N_7_-(G/A)( T/A) a(T/A) -3′). In this study, we systematically investigated the regulation of *Lp*lttR in *L. plantarum* and revealed that *Lp*LttR regulated different genes and performed different functions in different species. These findings deepened our understanding of the regulatory mechanisms of LttRs and provided a theoretical basis for the metabolism and regulation mechanisms of *L. plantarum.*

## Methods

### Strains

The strains and plasmids used in the present study were listed in Table 4. *L. plantarum* ATCC BAA-793 and the derivate strains were cultured in MRS medium at 37 °C under aerobic conditions. *E. coli* BL21(DE3) was used for the expression and purification of *LpLttR*. It was cultured in LB medium at 37 °C, 200 rpm. The process of protein expression and purification has been described in a previous study [8].

**Table 4:** Strains and plasmids used in this study

Strain or plasmid	Characteristic	Source or reference
*Strains*		
*Lactiplantibacillus plantarum* ATCC BAA-793	Wild type	[25]
*Lactiplantibacillus plantarum* ATCC BAA-793 Δ*LplttR*	*LplttR deletion mutant strain*	[8]
*Lactiplantibacillus plantarum* ATCC BAA-793/pIB184-*LplttR*	*lttR* overexpression strain, carrying pIB184 -*LplttR*	[8]
*E.coli* BL21(DE3)	Expression strain	Novagen
*Plasmid*		
pET28a-*LplttR*	pET28a derivate carrying *LplttR*	[8]
pIB184 -*LplttR*	pIB 184 carrying *LplttR* for gene overexpression	[8]
pLCNICK- *LplttR*	Used for *LplttR* deletion	[8]

### RNA extraction

The WT and Δ*LplttR* strains were collected during the exponential growth period with three biological replicates. The bacteria were sent to Majorbio Bio-Pharm Technology Co., Ltd (Shanghai, China) for subsequent transcriptome sequencing. TRIzol® Reagent (Invitrogen) was used to extract the total RNA according to the manufacturer’s instructions. Agilent 2000 was used for the RNA quality determination, and Nanodrop2000 (NanoDrop Technologies) was used for the quantification. The integrity of RNA was assessed by agarose gel electrophoresis. Only high-quality RNA samples (OD260/280 = 1.8 ~ 2.0, OD260/230 ≥ 2.0, RIN ≥ 6.5, 23S:16S ≥ 1.0, Concentration ≥ 100 ng/μl, and total amount of RNA ≥ 2 μg) were used for subsequent library construction.

### Library construction and transcriptome sequencing

The TruSeq™ Stranded Total RNA Library Prep Kit from Illumina (San Diego, CA, USA) was used to construct the library for the experiment. After removing rRNA and adding fragmentation buffer, mRNA was randomly broken into small fragments of approximately 200 nt. Under the action of reverse transcriptase, one-strand cDNA was synthesized using random primers and mRNA as templates. For the second strand synthesis, dUTP was used instead of dTTP to form the base of the second strand of cDNA containing dTTP. Before PCR amplification, the second strand of cDNA was digested with the UNG enzyme so that only the first strand of cDNA was included in the library. Finally, Illumina Hiseq × 10 (2 × 150 bp read length) was used for sequencing. Processing of the original images to sequences, base-calling, and quality value calculations were performed using the Illumina GA Pipeline (version 1.6), in which 150 bp paired-end reads were obtained.

### Bioinformatics analysis

The data generated from the Illumina platform were used for bioinformatics analysis. All the analyses were carried out using the I-Sanger cloud platform (www.i-sanger.com) from Majorbio Bio-Pharm Technology Co., Ltd (Shanghai, China). Sequencing reads were compared to those in the Rfm database. The accession number of the reference genome was GCF_000203855.3. The transcriptome sequencing raw data in fastq format raw reads were deposited in the National Center for Biotechnology Information Sequence Read Archive (NCBI SRA) database (accession number: PRJNA751435).

### Protein-DNA interaction

The regulatory regions of the target genes were amplified by PCR using the primers listed in Table 5. To label the DNA with biotin, a second PCR reaction was performed using a universal biotinylated primer (5′-biotin-AGCCAGTGGCGATAAG-3′). The PCR products were purified using a PCR purification kit (Shanghai Generay Biotech). The quality and concentration of the biotin-labelled DNA probe was analyzed using 1% agarose gel electrophoresis and a Nanodrop 2000 spectrophotometer (Thermo Fisher Scientific), respectively.

**Table 5:** Primers used in protein-DNA interaction

Primers	Sequence (5’-3’)
0057-S	AGCCAGTGGCGATAAGTTGGCATTTGCTGGTTC
0057-A	AGCCAGTGGCGATAAGGTTCATTCACGTCAACGC
P0202-S	AGCCAGTGGCGATAAGCGTGTTGATGTAAAATAACTTG
P0202-A	AGCCAGTGGCGATAAGTTTCTTTTGCCATTTGTTAT
P0203-S	AGCCAGTGGCGATAAGCTTAGAACGCAAAATATGAT
P0203-A	AGCCAGTGGCGATAAGTAAAATACCACCTACCAAAT
3533-S	AGCCAGTGGCGATAAGTTTTAAAATACGCTCCTGAG
3533-A	AGCCAGTGGCGATAAGGCAGGTTGGTTCATGAGA
3151-S	AGCCAGTGGCGATAAGACTGCCAACAATCACATC
3151-A	AGCCAGTGGCGATAAGTTTCATAAAATATTCCTCCA
2768-S	AGCCAGTGGCGATAAGTACAAACTATCAACCATAATTCG
2768-A	AGCCAGTGGCGATAAGATGGCGAGTTCCTTTCG
1745-S	AGCCAGTGGCGATAAGAGCCGTTGTTGGAATGC
1745-A	AGCCAGTGGCGATAAGGATTGCCTCTGTCATAGTCTG
0302-S	AGCCAGTGGCGATAAGCGGCTAATTTTCATCATTAG
0302-A	AGCCAGTGGCGATAAGGGTTTTTGATCTTCATAATAATT
2920-S	AGCCAGTGGCGATAAGCCATGCCACTAGCAACG
2920-A	AGCCAGTGGCGATAAGGTGTGTTTTAAATCCATAGGC

The specificity of binding and affinity constant of *Lp*LttR to the promoter of target genes were determined using the Octet system (Octet, ForteBio, USA) based on bio-membrane interference technology, according to the previous study [24]. The samples were added to the 96-well plates in a total volume of 200 μL. The reactions were performed at 37 °C with shaking at 1000 rpm. After loading with the biotinylated DNA, the streptavidin biosensors were transferred to His-LplttR solutions to associate, and then moved to running buffer to dissociate. The obtained data were processed by Octet Data Analysis version 7.0 using a 1:1 binding model.

### RNA preparation and RT-PCR

*L. plantarum* and the derivate strains in the exponential growth period were collected by centrifugation at 4 °C. The total RNA was prepared and analyzed by qPCR as previously described [8]. The primers used for RT-PCR were listed in Table 6. Each PCR condition was performed in triplicate on the LightCycler 96 qRT-PCR system (Roche Diagnostics, Switzerland). The PCR procedure was as follows: 95 °C for 5 s, followed by 40 cycles of denaturation at 95 °C for 5 s and annealing at 60 °C for 30 s. 16S rRNA was used as the internal control. The obtained data was analyzed using analytical LightCycler 96 system, and the 2^−ΔΔct^ method was used to calculate the transcriptional fold changes.

**Table 6:** Primers used in qRT-PCR

Primers	Sequence (5′–3′)
0057-qPCR-s	ACTTTGGTCCCAGAACG
0057-qPCR-a	GACGAGCGATGATAGGC
P0202-qPCR-s	TGTTTGCGATTTGATTG
P0202-qPCR-a	GCCATACTTGCGTTCCT
P0203-qPCR-s	GAGCGTTTAATAGTGTTC
P0203-qPCR-a	TTAGCAAGCCCGTCATC
3533-qPCR-s	ACGGTACGATTTGCTTG
3533-qPCR-a	CTGCGATGAACATTGAGA
3151-qPCR-s	ACAAGGGAAAGCTGATC
3151-qPCR-a	AGCACTGTTAGCCGTAA
2768-qPCR-s	CTTGCTTTGCCTTGTCC
2768-qPCR-a	GTTGCCGTAAATAAGTTGAT
3153-qPCR-s	GCCATTCAAGATTACGA
3153-qPCR-a	TGATAGGTGCAGATAAGG
1745-qPCR-s	CGCTGGTTGCAGGAATA
1745-qPCR-a	GCACCGAACGGAGTAAG
0302-qPCR-s	ATTAAGCCAAATGCAATCAC
0302-qPCR-a	AAACCATAGGCACCAGA
2920-qPCR-s	CACTGCTTGTCGGGTTA
2920-qPCR-a	ATGGGAATGTTGCTTGAT
qPCR-16S-F	CACATTGGGACTGAGACACGG
qPCR-16S-R	CGATGCACTTCTTCGGTTGAG

## Data Availability

All data analyzed in this study are included in this published article.
